# Opioid Use in Patients With Sickle Cell Disease During a Vaso-Occlusive Crisis: A Systematic Review

**DOI:** 10.7759/cureus.21473

**Published:** 2022-01-21

**Authors:** Hadia Arzoun, Mirra Srinivasan, Isra Sahib, Jack Fondeur, Lisbeth Escudero Mendez, Raneem K Hamouda, Lubna Mohammed

**Affiliations:** 1 Internal Medicine, California Institute of Behavioral Neurosciences & Psychology, Fairfield, USA; 2 Pathology, California Institute of Behavioral Neurosciences & Psychology, Fairfield, USA

**Keywords:** voc, vaso-occlusive crisis, opioid medication, sickle cell anemia, scd

## Abstract

Sickle cell disease (SCD) affects the red blood cells, which become sickle-shaped, leading to their adhesion to vascular walls, impeding blood flow and causing the unpredictable, abrupt onset of intense pain episodes in the form of vaso-occlusive crises (VOC) as well as affecting multiple organ systems. The primary aim of this review is to assess the effectiveness of opioid analgesic intervention for pain management in sickle cell disease during an acute painful crisis. A literature search was done electronically on PubMed Central (PMC), PubMed, and Google Scholar databases. The reports included in the study were from 2010 to 2021, and the bibliographies of retrieved studies are included in the references. This systematic review was undertaken as per the Preferred Reporting Items for Systematic Review and Meta-Analysis. This study included reports discussing opioid analgesics in SCD patients during VOC in different settings. After extensive research, there were no clear current opioid treatment patterns described, and our conclusion suggested conducting more evidence-based research to improve the quality of VOC management and outcome.

## Introduction and background

Sickle cell disease (SCD) is the most common inherited blood disorder that prevails in a specific population worldwide, primarily African Americans. In the United States, it is estimated to affect over 100,000 people, and about one out of every 365 African-American births is estimated to be living with sickle cell disease [1,2]. The most common clinical hallmark of SCD that dramatically impacts the health system, has been costly, and led to possible recurrent emergency department (ED) visits is the vaso-occlusive crisis (VOC) or vaso-occlusive event (VOE). Acute painful crisis (APC) is a consequence of microcirculation occlusion by sickle hemoglobin-containing red blood cells (RBCs), leading to ischemia, inflammation, and subsequent tissue damage [3]. These pain crises are often recurrent and unpredictable forms of acute pain [4]. The Cooperative Study of Sickle Cell Disease defines an acute pain crisis as an episode of pain in the extremities, back, abdomen, or head lasting more than two hours that leads to a clinic visit that cannot be explained by any etiology other than SCD. As severity varies, this excludes some pain episodes that can be manageable at home, chronic pain, as well as other multiple etiologies that can lead to acute painful complications of SCD such as acute chest syndrome, dactylitis, osteomyelitis, priapism, and right upper quadrant syndrome [5]. VOE can be diagnosed after all possible causes of pain are ruled out through clinical examination and other modalities of diagnosis [6]. Painful VOE can lead to a more profound illness and significantly affect health-related quality of life in SCD patients [7]. Multiple epidemiologic data indicated that up to five percent of SCD patients can have three to ten episodes of severe pain every year [8].

The majority of acute moderate-to-severe painful vaso-occlusive attacks are managed in the hospital using different pain management strategies, including non-opioids as well as opioid medications. Adequate pain management is by far the most important goal for these patients [6]. Potent analgesics in the form of parenteral opioids, such as intravenous morphine or hydromorphone, are considered the standard of care for acute, severe VOE. Moreover, the route, dose, and selection of a specific type of opioid vary significantly among SCD patients as the severity of the VOC event varies [9]. Other quick and pain-free methods that can result in more rapid and effective pain control and allow higher patient satisfaction include administration of intranasal route fentanyl [10].

Furthermore, as most treatments of VOC are initiated during the ED encounter as being considered the first point of care utilization, little is known regarding the pattern of opioid usage during VOC events and its effect in the long run, especially in the era of the opioid epidemic and narcotic addiction.

Moreover, healthcare providers are not adequately informed on how variable and intense these painful VOC can be and what should be initially done for managing them as they often view SCD patients as "opioid medication/drug seekers" due to the stigma associated with the need to control their pain with potent opioids and concerns regarding addiction [11]. Overall, considering suboptimal pain management of VOC in SCD patients can lead to the development of opioid tolerance as well as chronic pain with the possibility of developing neuropathic pain and a chronic need for narcotic medications [12]. Therefore, the American Pain Society and the American Academy of Pain Medicine advocate tailoring opioid analgesic therapy based on individualized risk assessment [13]. 

The primary objective of this systematic review will be to evaluate the pattern of opioid use in patients with SCD during a VOC and address if using narcotic analgesics to control and improve pain adequately will result in opioid-seeking behavior. Given the paucity of data, it also highlights the need for a deeper understanding of the clinical complexity of the disease and the role of using opioid analgesics with the need for further research and clinical correlation to set high-quality evidence for acute severe VOC management.

Methods

In this systematic review, the Preferred Reporting Items for Systematic Reviews and Meta-Analysis (PRISMA) 2020 standards and principles were followed [14].

Search Strategy

Three databases, namely, PubMed, Google Scholar, and PubMed Central (PMC), were extensively explored electronically, using the appropriate traditional keywords and medical subject headings (MeSH) keywords to accurately include all the potentially relevant articles that have sickle cell anemia and the role of opioid use in a vaso-occlusive crisis. The keywords used include SCD, sickle cell anemia, opioid medication, vaso-occlusive crisis, and VOC. The Boolean scheme was also used to precisely traditional and MeSH keyword searches and was employed in PubMed. All articles were retrieved, and only articles and subject headings relevant to this article were used.

Inclusion and Exclusion Criteria

This article only included randomized control trials, systematic reviews, and other published articles from 2010 to 2021. Among all the studies included, the articles selected were related to patients with SCD, vaso-occlusive crisis, and opioid medication use in different settings, including ED, and during hospitalization. Our eligibility criteria were based on population, intervention, comparison, outcomes, and study criteria (PICOs). Only studies published in the English language were included in this study. The review excluded studies that focused on chronic opioid use, pregnant women with SCD, and the use of other pain medications.

Data Extraction

Two researchers selected and extracted data autonomously, including all articles relevant to our inclusion and exclusion criteria, intervention used, and outcome combined. 

Quality Assessment and Analysis of the Studies

For traditional papers, the Scale for the Assessment of Narrative Review Articles (SANRA) was implemented. The score of each criterion was reported as having a score of more than nine. Similarly, the PRISMA checklist was used for quality appraisal of the systematic reviews. We evaluated the methodological quality of the research papers through this method, with a score of 23 and above set as the benchmark for inclusion.

Results

**Figure 1:**
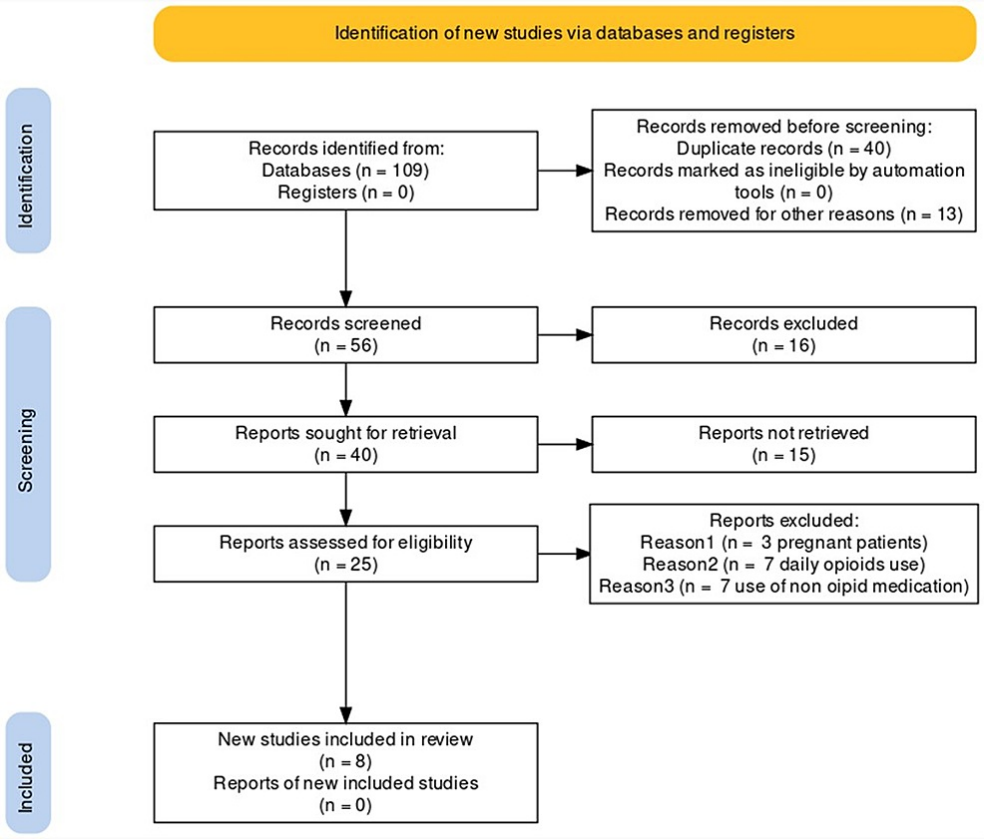
PRISMA 2020 flow diagram. PRISMA: Preferred Reporting Items for Systematic Reviews and Meta-Analysis.

A total of 109 articles were identified from different search tools using the specific search strategies identified in the keywords. Out of 109 articles, 34 were from PubMed, 25 were from PMC, and 50 were obtained from Google Scholar; 56 articles remained following the removal of 40 duplicate reports, and 13 were removed for other reasons. The remaining 56 articles were filtered according to the relevance extracted from the abstract for the title and content, after which 16 articles were excluded from this study. After applying inclusion/exclusion criteria with a full-text screen, 15 articles were excluded, and 17 articles were excluded during data extraction. The final eight reports were found relevant based on the eligibility criteria. A comprehensive PRISMA flow chart is shown in Figure 1 [14].

Table 1 summarizes each of the selected articles that are included in this review.

**Table 1:** Summary of included studies. RCTs: randomized controlled trails; APC: acute painful crisis; ED: emergency department; IV: intravenous; SCD: sickle cell disease; NHLBI: National Heart, Lung, and Blood Institute; ACU: acute care unit; VOC: vaso-occlusive crisis.

Author	Year of publication	Selection criteria	Study design	Finding/conclusion
Ballas et al. [3]	2012	Classification of sickle cell pain based on the pathophysiologic process.	Literature review	Managing sickle cell pain should be based on specific pathophysiologic mechanisms, not following other guidelines that treat non-sickle pain syndromes.
Dunlop et al. [15]	2014	Assessing the effectiveness of different pharmacological analgesic interventions for pain management in sickle cell disease from identified nine RCTs.	Systematic review	Limited evidence was found for analgesic interventions in acute pain crises. One study suggested no difference in the efficacy of using sustained-release oral and parenteral morphine, suggesting using oral morphine for acute pain management.
Telfer et al. [16]	2014	Assessing the possibility of using alternatives to opioid analgesics in adult patients during APC in the ED.	Literature review	A need for clinical studies to evaluate the efficacy and safety of other analgesics, such as oxycodone and fentanyl in comparison with morphine as well use of other routes of drug administration rather than IV route could result in more effective pain control as well as an overall reduction in opioid requirement and short hospital stay.
Gupta et al. [17]	2015	Effect of morphine use in the treatment of SCD pain.	Literature review	Morphine use may exacerbate pre-existent organ damage as well as lead to the development of new pathologies.
Telfer et al. [5]	2017	Review the problems with current models of treatment.	Literature review	The lack of a standardized protocol for managing an acute pain episode suggests adopting an integrated protocol to treat acute pain promptly.
Masese et al. [18]	2019	Barriers and facilitators to care in SCD patients in central North Carolina by ED providers.	Cross-sectional observational study	Half of the health care providers utilized individualized dosing protocol for sickle cell pain, whereas only a third of the providers were aware of the NHLBI recommendations.
Molokie et al. [19]	2020	Comparison between ACU vs. ED to address adult pain with opioid use.	Retrospective, observational study	Providing higher dosing of opioids for acute painful episodes in adults with SCD in ACU was associated with improved pain outcomes and decreased hospitalizations compared to ED.
Osborne et al. [11]	2021	Hospitalized adults with SCD who were prescribed opioids to treat VOC.	Systematic review	Hospitalized VOC patients were treated with opioids, but no uniform method of opioid administration was provided.

## Review

VOC is a leading cause of morbidity and a common reason for ED visits, which leads to subsequent hospitalization among patients with SCD. Opioid analgesics remain the treatment of choice for alleviating the acute and severe pain associated with VOC. The following sections discuss the pathophysiology of VOC, different opioids used in the management of acute pain crises in the ED and during hospitalization, and lastly, the potential adverse effects that might develop from opioid use.

Pathophysiologic events in acute VOC in SCD patients

**Figure 2:**
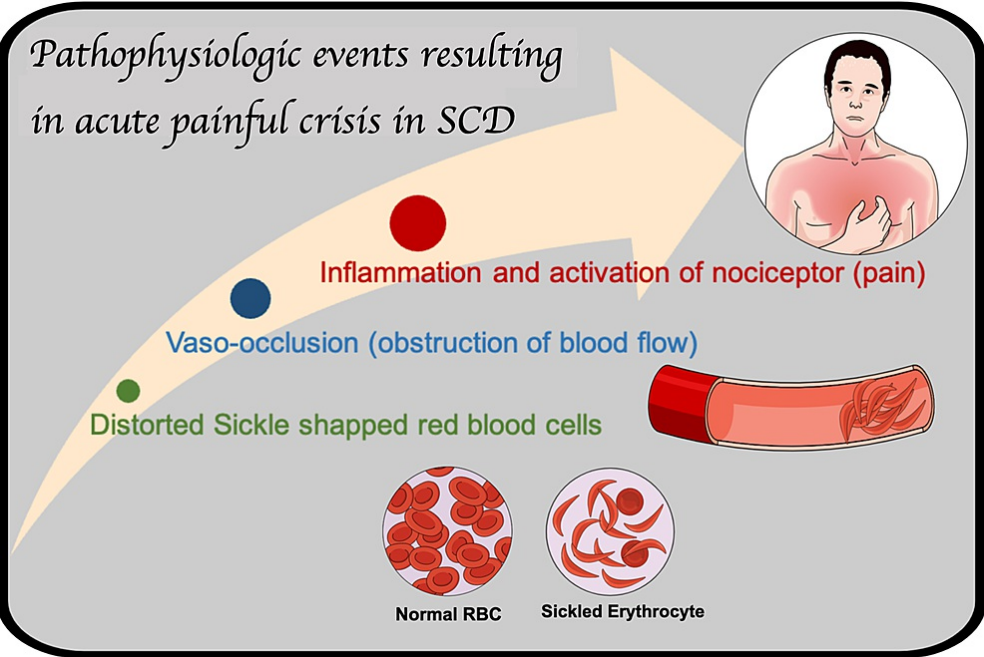
The pathophysiologic events in VOC. Figure created in mind the graph platform. SCD: sickle cell disease; RBC: red blood cell; VOC: vaso-occlusive crisis.

The most frequent presentation of SCD is an acute pain crisis, which requires a clinic visit or hospitalization. Multifactorial processes can trigger VOC in the form of patient-related or environmental factors or both, including infection, fever, dehydration, acidosis, exposure to temperature extremes, humidity, and pain itself, which can precipitate painful crises [20]. These pain episodes occur from the clustering of sickle-shaped red blood cells into the microcirculation, causing blood flow occlusion with impairment of oxygen supply, leading to infarction/reperfusion injury, inflammation, and subsequent tissue damage [5]. This inflammation is associated with the release of different chemical mediators that cause the activation of different nociceptive neural receptor pathways in different parts of the nervous system. These APCs are usually first diagnosed at the emergency department once other etiologies of pain are excluded. On average, each painful episode lasts for four to five days but sometimes continues for up to a week to subside [6]. Figure 2 depicts the pathophysiologic events that lead to the formation of acute painful crises [5].

Opioids are a widely used and acceptable form of analgesic for treating acute and severe pain. In 2014, the National Heart, Lung, and Blood Institute (NHLBI) released guidelines in an effort to improve outcomes for SCD patients during painful VOEs. They set recommendations for assessing APC patients through level two triage, administering IV opioids within 60 minutes of arrival to the ED, as well as implementing an individualized dosing protocol for additional analgesics if needed [18].

In a study comparing the NHLBI-recommended opioid dosing strategies for acute SCD pain in the ED, using a patient-specific protocol by employing patient-controlled analgesia (PCA) showed a significant reduction in pain scores, led to lower hospital admission rates and resulted in an acceptable side effect compared to weight-based protocol patients [1]. Moreover, in a study comparing nine randomized controlled trials (RCTs) using the pharmacological treatment for acute and chronic pain in SCD, only one trial showed no significant difference in the efficacy of sustained-release oral versus intravenous morphine in the mean overall pain scores as well as the frequency/severity of adverse effects, thus suggested to consider using use oral morphine for acute pain [15]. The amount of opioid requirement depends on the pharmacokinetics and pharmacodynamics of the opioid, subjective pain score and prior opioid needs, and presence or absence of a history of chronic opioid use. Opioid delivery by PCA to manage VOC is associated with a shorter length of hospital stay, decreased total opioid consumption, and better pain control [21]. This is in contrast to a study conducted by Al-Anazi et al. retrospectively compared two groups of SCD patients during the first 72 hours of admission for their pain intensity and pain relief using intermittent dosing of IV morphine versus PCA has shown that intermittent administration of IV morphine was more effective in managing VOC pain than PCA infusion and all patients underwent evaluation for adverse effects and results have shown no finding [22].

Morphine and hydromorphone have been used as the first-line intravenous opioid analgesics of choice for APC but are considered a less ideal form of analgesic as they raise multiple concerns. Fentanyl has also been described as an alternative to or in combination with morphine. It acts more rapidly in the CNS, is more potent with a shorter half-life than morphine, and can be administered through different routes [16]. 

In one study comparing opioid doses given to SCD patients during acute pain episodes in the acute care unit (ACU) were significantly lower than in the ED as this was explained by quicker administration of pain medication, use of higher mg/kg doses of morphine or hydromorphone for management of acute pain episodes of SCD than those used during ED management, which led to improvements in patients' quality of life and care, decrease the need for hospital admissions, and lower overall medical costs [19]. 

In another study, given careful attention to the severity of patient-reported pain, including assessing adequate pain relief following administration of the initial dose of morphine in the ED, should be used to help predict those who might need hospital admission for further pain management of VOE afterward with the initiation of PCA and prevent premature discharge. Furthermore, assessing the number of morphine doses prescribed in conjunction with overall reported pain scores can be used as an indirect measure of the physician's pain assessment for further management [23].

Therefore, using individualized opioid dosing with earlier administration of early analgesia with a maximum opioid dose would result in a shorter hospital stay [24]. Kim et al. evaluated outcomes in SCD patients during VOEs in an urgent care (UC) center following implementation of evidence-based practice standard care (EBPSC), including the meantime for administering the first analgesic dose, the mean length of stay, and overall patient’s satisfaction, and concluded that implementing EBPSC had resulted in improvement in the management of VOEs as well as provided patients with positive experiences [25]. 

Potential adverse effect following the use of narcotic analgesics in the treatment of VOC

The pharmacology and multi-organ adverse effects of opioids remain good areas of investigation due to a scarcity of clinical studies and considering the overall safety and efficacy of different opioids in the management of acute pain crises. Thus, understanding potential side effects and the need for adequate patient evaluation and ongoing monitoring is warranted. Gupta et al. suggested that as morphine has a multi-organ effect, this might result in exacerbating organ-specific pathological events as well as causing pathology by itself [17].

The documented acute adverse effects of morphine, described in a literature review done by Ballas et al., include overdose, respiratory depression, excessive sedation, altered mental status, nausea, vomiting, pruritus, and bowel habit changes in the form of constipation. These adverse effects are due to morphine's side effects on both neural and non-neural targets, as well as being described as the most histaminergic opioid and are excreted primarily in the urine [3]. In addition to this, as morphine is metabolized by liver glucuronidation, dosage adjustment should be considered in patients with liver dysfunction [8]. One study concluded that with each VOC event, there is an expected 9.5% increase in opioid prescription use [4]. Long-term opioid use is associated with the risk of abuse potential, leading to physical tolerance, physical and psychological dependence, addiction, and possible opioid hyperalgesia [5]. Furthermore, the Darbari et al. study found there was an unexplained increase in morphine clearance by three to ten folds in patients with SCD during painful crises, leading to lower plasma levels than in individuals without SCD after exclusion of hepatic or renal dysfunction as well as recent opioid use for at least two weeks, which might explain the likely need for higher and more frequent dosing in SCD patients to achieve comparable plasma levels [26].

Moreover, there were no consistent data exists to address the type, method, and amount of opioid administration in hospitalized sickle cell patients during VOC with the need to implement multi-modal evidence-based strategies as well as educate health care personnel in the management of VOC and the resulting SCD pain [11]. For these reasons, many factors have been identified to influence patient care and are associated with suboptimal analgesia and have been described as barriers to adequate care for patients during ED visits, including health care providers' fear of possible drug-seeking behaviors as well as frequent ED visits for pain management [27]. In the surveys conducted by Masese et al., ED providers in North Carolina showed a lack of implementation of the NHLBI recommendation for SCD care and advocated the use of individualized pain control. Moreover, barriers to care were described as patient behaviors and being described as “frequent fliers” or being stigmatized as “drug seekers” with a concern about addiction as well as provide attitude toward SCD patients [18].

Opioid analgesics seem to be widely used in patients with SCD, mortality data in those patients is actually related to the complications of the disease itself rather than opioid use. The problem relies on the misuse of opioids [28]. Al Zahrani et al., retrospectively evaluated SCD patients who had opioid use disorder (OUD) who were established in a multidisciplinary team (MDT) involving multiple educational activities for caregivers and patients; establishing a palliative/pain clinic and an SCD addiction clinic, as well as implementing an adequate opioid prescription tracking system, re-assessment of the method of pain assessment to avoid unnecessary interventions and tracking opioid prescriptions in both the ED and during hospital admission resulted in a significant reduction in both the numbers of ED visits and hospital admissions, as well as a dramatic decrease in the use of parenteral opioid consumption [29].

Limitations

The limitations to be considered in this systematic review are the relatively small number of articles selected and included in this review. In addition, no clinical trials and evidence-based data might be one of the limiting factors. Moreover, the included articles were different in study design and some were narrative reviews. Finally, this study didn't review reports that describe other therapies, including non-opioid analgesics used during the crisis episode.

## Conclusions

No studies addressed the immediate and long-term adverse effects of using opioid medications during acute, severe, painful sickle cell disease attacks. Given the paucity of data, there is a lack of evidence-based studies that suggest the development of frequent or severe adverse effects, including opioid addiction, during VOCs. Furthermore, no high-quality studies describe the optimal pain control regimen in adult SCD patients, and there is no recommendation for a specific agent to be used. Overall, the need for information about opioid dosage, route, and selection should be individualized based on the patient's pain score and the degree of pain relief following opioid administration to develop dosing guidelines to improve outcomes of VOC. In order to alleviate the current stigma of opioid use in the era of the opioid epidemic in concern to the use of non-standardized dosages of opioids to relieve VOC, continuous evidence-based research is needed.
